# Squalene epoxidase is a *bona fide* oncogene by amplification with clinical relevance in breast cancer

**DOI:** 10.1038/srep19435

**Published:** 2016-01-18

**Authors:** David N. Brown, Irene Caffa, Gabriella Cirmena, Daniela Piras, Anna Garuti, Maurizio Gallo, Saverio Alberti, Alessio Nencioni, Alberto Ballestrero, Gabriele Zoppoli

**Affiliations:** 1 J.-C. Heuson Breast Cancer Translational Research Laboratory, Institut Jules Bordet, Brussels BE; 2 Department of Internal Medicine (Di.M.I.), University of Genova, Genova IT; 3 Unità di Patologia Oncologica CeSI, Università “G. D’Annunzio”, Chieti Scalo (Chieti) IT; 4 Istituto di Ricovero e Cura a Carattere Scientifico Azienda Ospedaliera-Universitaria San Martino and National Cancer Institute, Genova IT

## Abstract

*SQLE* encodes squalene epoxidase, a key enzyme in cholesterol synthesis. *SQLE* has sporadically been reported among copy-number driven transcripts in multi-omics cancer projects. Yet, its functional relevance has never been subjected to systematic analyses. Here, we assessed the correlation of *SQLE* copy number (CN) and gene expression (GE) across multiple cancer types, focusing on the clinico-pathological associations in breast cancer (BC). We then investigated whether any biological effect of SQLE inhibition could be observed in BC cell line models. Breast, ovarian, and colorectal cancers showed the highest CN driven GE among 8,783 cases from 22 cancer types, with BC presenting the strongest one. *SQLE* overexpression was more prevalent in aggressive BC, and was an independent prognostic factor of unfavorable outcome. Through SQLE pharmacological inhibition and silencing in a panel of BC cell lines portraying the diversity of *SQLE* CN and GE, we demonstrated that SQLE inhibition resulted in a copy-dosage correlated decrease in cell viability, and in a noticeable increase in replication time, only in lines with detectable *SQLE* transcript. Altogether, our results pinpoint *SQLE* as a *bona fide* metabolic oncogene by amplification, and as a therapeutic target in BC. These findings could have implications in other cancer types.

The gene *SQLE*, found on chromosome 8q24.13 in humans^1^,^2^,^3^, encodes squalene epoxidase, one of the key enzymes in the later stages of cholesterol synthesis^3^,^4^,^5^,^6^,^7^. SQLE catalyzes the oxidation of squalene to 2,3-oxidosqualene^5^,^6^,^8^, downstream HMG-CoA reductase (*HMGCR*), the target of the statin class of cholesterol-lowering agents^9^. *SQLE* expression is almost ubiquitous in humans, with higher levels in skin, gastrointestinal mucosa, and central nervous system, and lower expression in skeletal muscle^10^. *SQLE* homologs can be found in several eukaryotic organisms. Indeed, fungal squalene epoxidase is inhibited by antimycotic agents, in use either topically or by oral administration since the 1990s (reviewed in^11^). Of interest, some of those drugs also block the activity of SQLE at high concentrations in *in vitro* models^12^,^13^. Several investigators actively pursued the development of potent, selective human SQLE inhibitors as a feasible way to lower cholesterol^14^,^15^,^16^. However, the description of potential side effects such as skin rash^18^ or peripheral demyelination^19^ in animal models and the widespread adoption and efficacy of statins^17^ halted the clinical development of SQLE inhibitors and their use in humans^11^,^20^: this happened in a possibly premature way and in spite of promising results^21^. As a consequence, the interest in SQLE-targeted drug development progressively faded in later years, and to our knowledge, no clinical study has been conducted to this day.

With the advent of –omics technologies and high-dimensional cancer multilayered analyses (exemplified by^22^), several works were published, identifying cancer genes with a potential for copy-number (CN) driven overexpression. In breast cancer, research results highlighted the high correlations between CN and gene expression (GE) of unsuspected and widely known oncogenes by amplification, such as *ERBB2* (coding the ERBB2 protein, also known as Her2) and *MYC*^23^,^24^,^25^,^26^,^27^,^28^,^29^,^30^,^31^. Scarce interest was dedicated, however, to the not infrequent appearance of *SQLE* among the top CN-GE correlating genes in several of those works (e.g., in^23^,^24^,^26^,^30^). This was possibly due to *SQLE* relative proximity to *MYC*^2^, and to the then-prevailing focus on aberrations in the targetable kinome, a major topic of oncological research in the 2000s^32^. Few articles from mid-size datasets described the potential role of SQLE overexpression in the definition of a prognostically unfavorable stage I-II estrogen receptor positive (ER+) breast cancer subgroup^33^ or in Afro-American luminal-A breast cancer patients^34^, and the identification of clusters of breast tumors characterized, among other alterations, by *SQLE* CN amplification and overexpression^25^, or by its aberrant methylation and expression patterns in concomitance with *MYC* amplification^35^. No further systematic, large-scale effort was however undertaken as a follow-up of those isolated observations.

In the present work, we set up to exhaustively analyze the correlation of *SQLE* CN and GE across multiple cancer histological types, Here, we provide the first, systematic, large-scale assessment of the prevalence and interaction of *SQLE* CN amplification with its GE variation in breast cancer and other tumor types in humans. We subsequently focused our efforts on the association of clinical and pathological factors with *SQLE* in breast cancer as well as on the potential prognostic relevance of *SQLE* in that disease. Finally, we investigated whether any biological effect of SQLE inhibition could be demonstrated from experiments performed in thoroughly characterized breast cancer cell line models.

## Results

### SQLE gene expression shows a high correlation with its locus copy number in breast cancer

**Figure 1:**
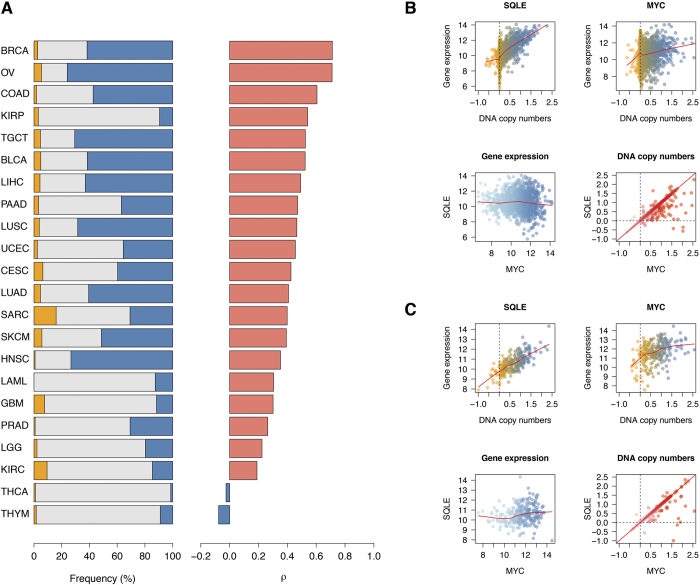
Correlation of *SQLE* copy number and gene expression is highest in breast and ovarian cancers. All cancer histotypes with at least 100 cases collected by the TCGA were assessed for the presence of *SQLE* copy number gains and losses (blue and orange respectively, left bar chart (**A**), and *log2* ratios were correlated with normalized *SQLE* gene expression using the Spearman’s correlation coefficient (ρ value on the *x*-axis, decreasing from highest to lowest, right bar chart (**A**). *SQLE* copy number/gene expression correlation was invariably higher than for *MYC*, which is physically closely located on chromosome 8q, in both breast (topmost panels, (**B**) and ovarian cancer (topmost panels, (**C**). Moreover, *SQLE* and *MYC* gene expression values were not correlated in those cancer types (bottom left B and C), whereas copy number values were almost identical due to the aforementioned chromosomal proximity (bottom right B and C). Topmost *y*-axes and bottom left *x*- and *y*-axes C and D: normalized *log2* gene expression intensity. Bottom right *x*- and *y*-axes C and D: *log2* ratios for copy number values. Full definitions of the TCGA acronyms can be found at https://tcga-data.nci.nih.gov/tcga.

To systematically assess which cancer histotypes showed an association between chromosome 8q24.13 locus CN, where *SQLE* resides^1^,^2^, and *SQLE* gene expression, we analyzed publicly avaimlable data generated by The Cancer Genome Atlas (TCGA), limiting our study to 22 cancer types with at least 100 assessed patients (n = 8,783 cases, see Table 1), and with available SNP6 arrays and RNA-sequencing data. Gains at the SQLE locus, defined according to the GISTIC2.0 algorithm^36^,^37^, presented the highest frequency in ovarian cancer and the lowest in glioblastoma multiforme (76% and 12% of affected patients respectively, see Fig. 1A, leftmost panel). 62% of breast cancer patients were characterized by gains at the *SQLE* locus. After correction for multiple testing, we found that 20 of the 22 cancer sets still presented a positive association between *SQLE* locus CN and *SQLE* GE, with a false discovery rate (FDR) < 0.001 (see Fig. 1A, rightmost panel). However, only breast, ovarian, and colorectal cancers showed a large effect size^38^ for that association, with breast cancer presenting the strongest correlation (ρ = 0.71, see Fig. 1C top left) since it also represented the largest TCGA dataset, with 1,178 collected cancer specimens. Ovarian cancer had a similar *SQLE* CN-GE correlation coefficient (ρ = 0.71, see Fig. 1D top left), followed by colorectal cancer with ρ = 0.61. Taken together, these data suggest that *SQLE* CN may have a tissue-dependent role in contributing to the variability of *SQLE* GE, and that breast and ovarian cancers show the tightest correlation between those two parameters. As a comparison, it is worth noting that a prototypical oncogene driven by copy number amplification, *ERBB2*, shows a ρ = 0.63 in the same TCGA breast cancer dataset where *SQLE* was assessed.

### SQLE and MYC are transcriptionally independent in breast and ovarian cancer, albeit residing in close proximity on chromosome 8

Since its cloning and characterization^39^, *MYC* has been one of the most studied cancer-related genes. It is physically located on chromosome 8q24.21, the cytoband immediately adjacent to the *SQLE* locus, towards the telomeric end^2^. We therefore explored *MYC* and *SQLE* associations in terms of GE and CN in the breast and ovarian cancer TCGA datasets (see Supplementary Table S1 and Supplementary Fig. S1 online). Although almost invariably *SQLE* and *MYC* loci presented identical or near similar *log2* ratio values (ρ = 0.96 in breast cancer and 0.89 in ovarian cancer, see Fig. 1C,D bottom right), *SQLE* and *MYC* transcripts were not correlated with each other (see Fig. 1C,D bottom left). Moreover, *MYC* showed a much lower CN-GE correlation coefficient than *SQLE* in both breast and ovarian cancers (ρ = 0.11 in breast cancer and 0.40 in ovarian cancer, see Fig. 1C,D top right). These observations may imply that the locus, spanning chromosome 8q23.13-q24.21 is a strong driver for *SQLE* expression in breast and ovarian cancer, explaining a relevant proportion of its variability across samples, whereas possibly other variables, in addition to copy dosage, may concur in determining *MYC* transcriptional levels in those two cancers. Finally, *MYC* transcript does not seem to behave as a transcriptional driver of *SQLE* expression in breast and ovarian cancer, as anticipated by the absence of *MYC* promoter elements upstream of *SQLE*^40^.

### SQLE is overexpressed in clinically aggressive breast cancer

**Figure 2:**
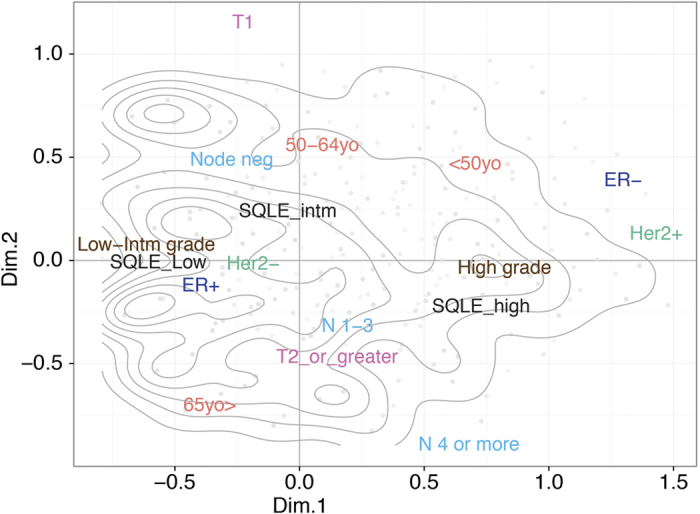
Multiple correspondence analysis (MCA) defines the structure of clinical and pathological associations with *SQLE* gene expression in breast cancer. *x*- and *y*-axes represent the first and second dimension (Dim.1 and Dim.2) of the MCA analysis performed on clinical and pathological data, as well as *SQLE* expression, from 1,663 breast cancer patients reported in the METABRIC dataset. The contour lines display areas of similar sample density, with smaller circles representing peaks in such density. Grey dots represent individual patients, with darker shades indicating more data points falling in the same MCA region. Variable categorical levels are automatically positioned according to their correlations with such dimensional reduction vectors and with each other. In particular, patients with high-grade tumors also show low *SQLE* expression levels (bottom right region), whereas the cluster of patients with *SQLE* low expresser tumors is also characterized by low and intermediate (Low/Intm) grade, Her2-, ER + cancers (center left region).

To explore the associations of *SQLE* expression with classical clinical and pathological variables in breast cancer, i.e. age, tumor size, number of positive lymph nodes (NPLN), histological grade, ER and Her2 status, we took advantage of the data generated by METABRIC, currently the single largest clinically annotated CN and GE breast cancer dataset publicly available^31^. Using multiple correspondence analysis (MCA), a multivariate statistical method similar to principal component analysis, but suited for categorical data^41^, we reduced our variables of interest to two dimensions, explaining the largest fraction of the variance characterizing the factors we considered from the METABRIC data. Clinical and pathological variables together with *SQLE* expression levels were projected as vectors in a space defined by those two dimensions (see Fig. 2). The position of the variable categories in this two-dimensional space reflects their mutual associations, with no *a priori* assumption on the underlying structure of the data. We observed that elevated *SQLE* expression lay in close proximity with high histological grade, and in the same spatial region where positive nodal status and larger (T2 or greater) T size were positioned. On the other hand, low *SQLE* expression clustered together with low/intermediate grade, ER + and Her2- status. Overall, this implies that patients developing less aggressive, ER+, Her2- breast cancer have lower *SQLE* expression levels, whereas aggressive tumors are more often characterized by high SQLE transcript values.

### High grade and Her2 + status are highly associated with SQLE overexpression

By univariable linear regression, we confirmed the associations observed by MCA. In particular, ER + status was positively associated with low *SQLE* expression (Δ *log2* expression = −0.36, 95% CI = −0.45–−0.26, P = 5 × 10^−13^), as were smaller tumors (Δ *log2* expression = −0.13, 95% CI = −0.21–−0.04, P = 0.0032). On the other hand, we found a significant association of elevated *SQLE* levels with high-grade tumors (Δ *log2* expression = 0.51, 95% CI = 0.43–0.58, P < 2 × 10^−16^), node positive cancers (Δ *log2* expression for NPLN greater or equal 4 vs. 0 = 0.17, 95% CI = 0.04–0.31, P = 0.0086 and NPLN 1–3 vs. 0 = 0.14, 95% CI = 0.03–0.24, P = 0.0072, overall P = 0.0011), and Her2 + neoplastic malignancies (Δ *log2* expression = 0.41, 95% CI = 0.30–0.54, P = 1.7 × 10^−11^). The largest effect sizes for those associations could be observed for histological grade and Her2 status, which indeed remained independently significantly associated with *SQLE* GE in a multivariable linear regression model including all the aforementioned clinical and pathological parameters (see Table 2). In summary, by both MCA analysis and classical statistical tests we could demonstrate that, in breast cancer, high *SQLE* GE characterizes clinically aggressive tumors (higher grade, larger size, and positive nodal status ones), and that *SQLE* tends to be overexpressed in Her2 + and ER- cases.

### CN alterations in the SQLE-MYC locus denote a poorer outcome in breast cancer

**Figure 3:**
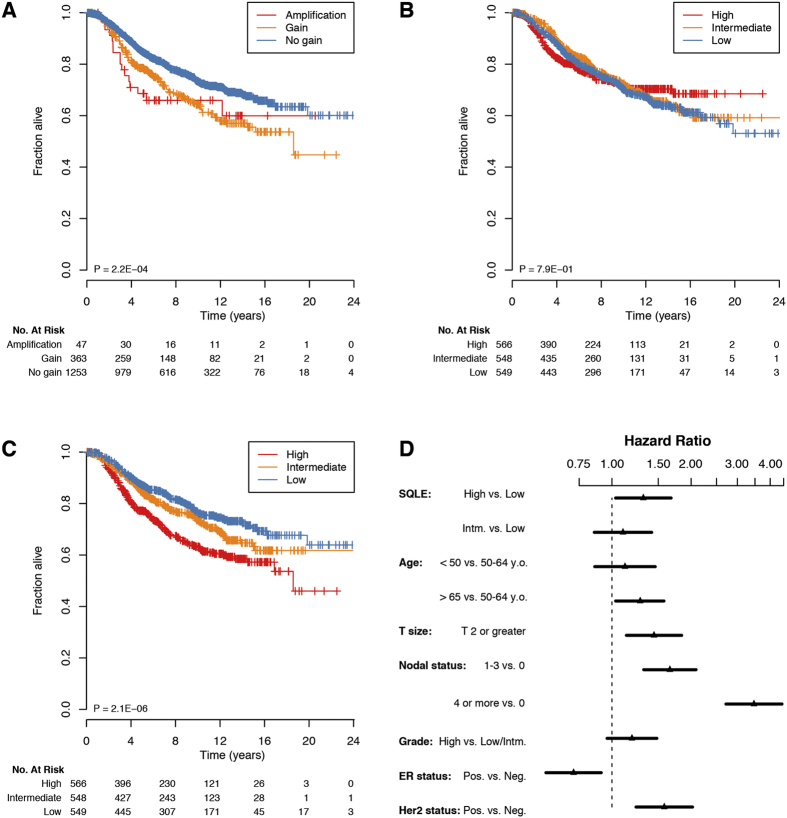
*SQLE* high expression is independently associated with unfavorable prognosis in breast cancer. Patients affected by tumors bearing chromosome 8q24.13-q24.21 (the region encompassing *SQLE* and *MYC*) gains or amplifications are characterized by poorer overall survival (OS), (**A**). However, whilst *MYC* expression levels have *per se* no prognostic value (**B**), *SQLE* overexpression is not only associated with worse OS by univariable analysis (**C**), but also maintains its independent prognostic value in a Cox proportional hazard model, including all classical clinical and pathological parameters, as shown in the forest plot depicted in panel (**D**) (HRs represented by black triangles and 95% CIs showed as horizontal black lines). Data are from the METABRIC set. P values in the bottom left area of Kaplan-Meier curves are obtained using the log-rank test. Panel D abbreviations: Intm. = Intermediate, y.o. = years old, NPLN = number of positive lymph nodes.

We subsequently assessed the prognostic value of chromosome 8q24.13-q24.21 CN alterations in the METABRIC dataset. Due to the extremely high correlation of *SQLE* and *MYC log2* ratio values in this set (ρ = 0.93), we used the mean of *log2* ratios for those two regions as a proxy for the CN status of chromosome 8q24.13-q24.21. Alterations at this locus purported a poor prognosis in breast cancer, with a significantly shorter overall survival in patients carrying CN gains or, with an even higher hazard ratio, amplifications (HR for gains = 1.51, 95% CI = 1.22–1.86, P = 0.0002; HR for amplifications = 1.67, 95% CI = 1.01–2.77, P = 0.0447; overall log-rank P = 0.0002, see Fig. 3A).

### SQLE overexpression, but not MYC overexpression, is an independently significant unfavorable prognostic biomarker in breast cancer

Next, we asked whether *SQLE* or *MYC* expression could bear any association with survival in breast cancer patients. To our surprise, there was no difference in survival rates in patients who developed tumors with low, intermediate, or elevated levels of *MYC* in the METABRIC dataset (P = 0.7945, see Fig. 1B). On the other hand, when stratifying patients by their *SQLE* GE values, we observed that the instantaneous risk of disease-specific death increased as a function of *SQLE* transcript abundance (HR for intermediate vs. low *SQLE* levels = 1.25, 95% CI = 0.98–1.61, P = 0.0754; HR for high vs. low *SQLE* levels = 1.79, 95% CI = 1.42–2.25, P = 9 × 10^−7^; overall log-rank P = 2 × 10^−6^, see Fig. 3C). Of even greater relevance, in a Cox proportional hazards regression model starting from all the classical clinical and pathological parameters (i.e., age, tumor size, NPLN, histological grade, ER, and Her2 status), as well as both *MYC* and *SQLE* GE values, only *SQLE* was retained, together with clinico-pathological parameters, as an independently significant variable for survival prediction in the final model (adjusted HR for the *SQLE* high vs. low expression strata = 1.32, 95% CI = 1.03–1.68, P = 0.0267; overall P of the final model = 0, see Table 3 and Fig. 3D). We intentionally did not include the integrative clustering subgroups^31^ or the PAM50 intrinsic subtypes^42^ as variables in our model: due to the strong independent prognostic value of those genomic classifiers, they would be retained at the cost of excluding more traditional parameters such as grade and ER status, which were the focus of this exploratory characterization of *SQLE* in breast cancer. To summarize, we demonstrated that *SQLE* overexpression is independently associated with an unfavorable outcome in breast cancer, even when taking into consideration classical clinical and pathological variables such as age, tumor size, nodal status, grade, ER, and Her2 status.

### Breast cancer cell lines show highly variable SQLE CN and GE

**Figure 4:**
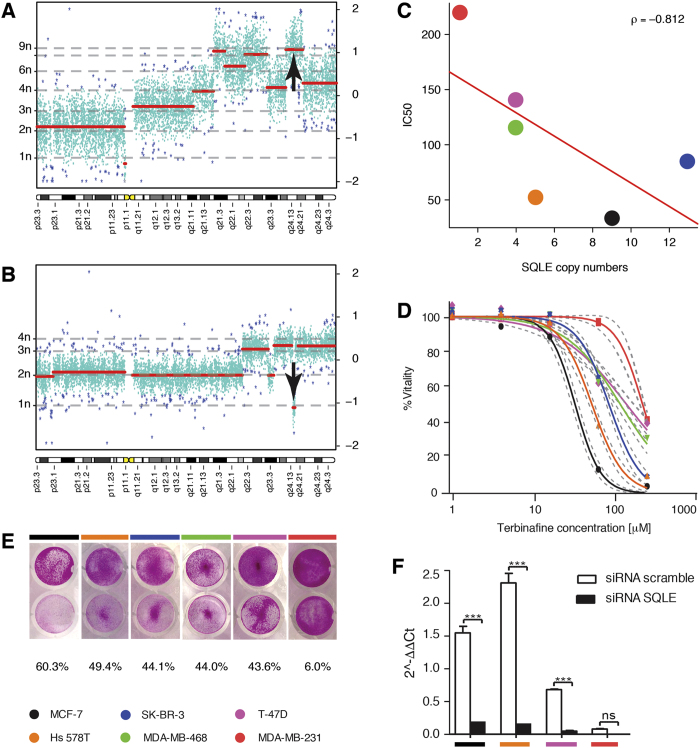
The cytotoxic action of the SQLE inhibitor terbinafine is associated with *SQLE* copy dosage in breast cancer cell lines. The chromosome 8 karyogram of breast cancer cell lines with the lowest (MCF-7) (**A**), and highest (MDA-MB-231) (**B**), IC_50_ for terbinafine are reported: along the *x*-axis are chromosomal coordinates, left *y*-axis shows the absolute copy number for segmented regions (thick red lines), and right *y*-axis reports *log2* ratios. Turquoise points are individual probes used for segmentation, whereas dark blue asterisks are array outliers. A vertical thick black arrow points toward SQLE locus in both karyograms. Terbinafine IC_50_ is anti-correlated (red line representing linear regression and ρ being Spearman’s correlation coefficient) with *SQLE* copy dosage (**C**), with a greater than 7-fold difference between least and most sensitive cell line (**D)**, dashed grey lines representing 95% confidence intervals). Sulforhodamine B coloration shows the residual cell mass upon 48 hours of terbinafine treatment (**E**). Silencing efficiency by *SQLE*-directed siRNA was always greater than 90% in treated lines (***P < 0.001, whiskers represent S.E.M.) (**F**). Color codes for cell lines are reported in the bottom left of the figure.

With the purpose of establishing an *in vitro* breast cancer cell line panel for *SQLE* characterization, we selected and acquired six breast cancer lines reported to present alterations in *SQLE* CN and GE levels from the CCLE^43^ or the NCI-60 cell line panel^44^, after assessing the data available through the cBioPortal for Cancer Genomics^45^. We then characterized those lines for ploidy and absolute *SQLE* CN value by array-CGH, and GE by qPCR (see Table 4, Fig. 4A,B, and Supplementary Fig. S2 online). SK-BR-3 and MCF-7 (see Fig. 4A) showed the highest levels of *SQLE* CN, each with more than 8 copies, through focal amplification of the chromosome 8q24.13 cytoband. Hs 578T presented the highest *SQLE* GE values and 5 copies of the gene through chromosome 8q gain, while MDA-MB-468 and T-47D were hypo-triploid, and were not characterized by *SQLE* focal alterations. MDA-MB-231 was unique, in that it behaved as a natural knockdown for *SQLE*, carrying only one copy of this gene and almost undetectable *SQLE* GE levels (see Fig. 4B). Altogether, the six breast cancer cell lines we selected were representative of the full spectrum of *SQLE* possible alterations, ranging from high-degree focal amplification, through arm-level gains with *SQLE* overexpression, to deletion with almost undetectable endogenous *SQLE* transcript.

### SQLE pharmacological inhibition decreases breast cancer cell line viability in a copy-dosage correlated way

Due to *SQLE* association with breast cancer aggressiveness, we hypothesized that its inhibition would lead to a decrease in cell proliferation. To control for potentially aspecific toxic effects of cholesterol biosynthesis inhibition, we assessed in parallel cell lines with differential levels of expression of *SQLE*. We therefore tested the viability of our six breast cell lines upon challenging with a SQLE inhibitor, terbinafine^46^. Terbinafine is used as an antifungal agent, since it inhibits fungal SQLE at plasma concentrations between 0.34 and 3.4 μM^47^. However, it also targets mammalian SQLE at higher concentrations^12^,^13^. Terbinafine indeed caused cancer cell demise in our breast lines, with an IC_50_ varying by almost an order of magnitude across the six assessed lines (see Table 4 and Fig. 4D,E). Of interest, terbinafine exerted its effect in an evident *SQLE* copy-dosage correlated manner (ρ = −0.81, P = 0.0499, see Fig. 4C). Moreover, Hs 578T showed a peculiar sensitivity to terbinafine, in spite of having a lower *SQLE* CN value than other lines. Not surprisingly however, Hs 578T was the highest SQLE expresser in our panel, and indeed also *SQLE* GE levels showed a trend for correlation with terbinafine IC_50_ in the six lines panel (ρ = −0.71, P = 0.1361). In summary, we demonstrated that pharmacological inhibition of SQLE effectively decreases breast cancer cell line viability in a copy-dosage correlated manner, suggesting that SQLE may be a treatment target in such disease, and that *SQLE* CN and/or GE increase could be used as predictive biomarkers of sensitivity to selective inhibitors of mammalian SQLE activity.

### SQLE silencing increases doubling time only in SQLE-expresser breast cancer cell lines

Since terbinafine may act at least partially in a non-specific manner at high concentrations^48^, we induced transient *SQLE* silencing in two lines with high endogenous *SQLE* transcript levels (MCF-7 and Hs 578T), one line with low but detectable *SQLE* (T-47D), and the cell line carrying a deletion in *SQLE*, MDA-MB-231. Silencing was highly effective, since it reduced *SQLE* GE by more than 90% at 24 h in all the three lines with detectable *SQLE* levels at baseline, compared to scrambled siRNA control (P < 0.001, see Fig. 4F). Upon *SQLE* silencing, all the three *SQLE* expresser cell lines showed an increase in their doubling times, by 47%, 17%, and 33% respectively, compared to cells treated with scrambled siRNA (see Table 4). MDA-MB-231, on the other hand, did not show any lengthening in its replication time. Taken together, our results demonstrate that targeting *SQLE* transcript by transcriptional silencing has an inhibitory biological effect, only in breast cell lines that exhibit endogenous *SQLE* transcription, with MDA-MB-231 acting as a natural negative control for our experiments. Moreover, our findings constitute a methodologically independent confirmation that the decrease in cell viability, observed in cell lines treated with terbinafine is indeed due to SQLE inhibition.

## Discussion

In the present article, we assessed the presence of CN and GE aberrations of *SQLE*, a key enzyme in the synthesis of cholesterol^3^,^4^,^5^,^6^,^7^, across more than 8,000 cases from 22 cancer types made available by the TCGA. We found that *SQLE* CN gains are frequent in several histologies, that *SQLE* GE is tightly correlated with its CN values, and that the strength of this association is tightest in breast cancer, followed by ovarian and colorectal cancer. *SQLE* CN-GE correlation appears to be systematically stronger in breast and ovarian tumors compared to the same association calculated for *MYC* in those cancer types, in spite of the close proximity of the two gene loci^2^, which results in similar CN values for both genes. By exploring *SQLE* correlations with clinical and pathological variables in METABRIC^31^ (the single largest, clinically annotated, publicly available CN/GE breast cancer dataset), we established that aggressive cases, defined by high histological grade, larger tumor size, nodal involvement, and by ER- and Her2 + disease, are characterized by *SQLE* overexpression. Moreover, we observed that *SQLE* overexpression, but not *MYC*, is independently significantly associated with unfavorable outcome in breast cancer, even after taking into account the above-mentioned clinical and pathological parameters. Finally, through SQLE pharmacological inhibition and *SQLE* transcript-directed silencing experiments in a panel of breast cancer cell lines portraying the diversity of *SQLE* CN and GE, we demonstrated that SQLE inhibition results in a decrease in cell viability that is highly correlated with *SQLE* copy dosage, and that induced reduction of *SQLE* GE levels causes a noticeable lengthening in replication time, only in cells with endogenous detectable *SQLE* transcript.

Altogether, our results pinpoint *SQLE* as a *bona fide* metabolic oncogene by amplification, as well as a therapeutic target in breast, and possibly, other cancer types, since it responds to major requirements to be considered as an oncogene of therapeutic relevance^49^: first, *SQLE* is frequently altered by CN gains in breast cancer, and *SQLE* GE appears to be tightly regulated by increases in the copy dosage of its gene locus; second, SQLE is a key enzyme in the synthesis of cholesterol, and several studies have pointed toward a therapeutic effect of cholesterol lowering in cancer, possibly by decreasing cholesterol bioavailability, altering cancer cell membranes, and through other mechanisms^50^,^51^,^52^; third, due to the CN-driven GE increase found only in cancer cells, *SQLE* may behave as an “oncogene by addiction”^53^ (as opposed for example to *HMGCR*, the target of statins, for which no recurrent aberrations have been described in tumors), such that mammalian SQLE inhibition may have a high therapeutic index, leaving healthy cells relatively unharmed by SQLE blockade, whilst provoking cell demise in *SQLE*-amplified cells only: one such notable example is the selective activity of anti-ERBB2 agents in *ERBB2*-amplified breast cancer, which has led to their successful adoption in clinical practice; last but not least, researchers have already developed potent and selective inhibitors of mammalian SQLE^14^,^15^,^16^, albeit with an entirely different scope than the one we foresee, i.e. as anti-cancer agents.

Of interest, several genes, apart from *SQLE* and *MYC*, located in the q arm of chromosome 8 may have an even tighter CN-GE correlation than *SQLE*, and be endowed with essential biological properties for cancer proliferation and survival. A notable example is *RAD21*, found in close proximity to *SQLE* on chromosome 8q24.11^2^. *RAD21* encodes a protein involved in DNA double-strand-break repair^54^, shows high-level amplifications in 37% of the TCGA breast cancer cases, and presents a significant transcriptional correlation with *SQLE* GE (ρ = 0.62) (data from http://www.cbioportal.org, accessed May 2^nd^, 2015). Our findings are not in contrast with a pleiotropic, multipronged impact of the amplification of this region. Notwithstanding, on the one hand a direct role of *SQLE* overexpression in promoting neoplastic growth was shown by the clear *in vitro* evidence we generated, that SQLE inhibition causes cell demise and slows replication: we have therefore a rationale to consider *SQLE* as an especially relevant cancer-gene belonging to that region. On the other hand, it is entirely possible that several proteins encoded in the same chromosomal region cooperate to determine a more aggressive phenotype in cancer: indeed, it has been demonstrated that genes with a common final function can be physically clustered in the same genomic interval (see^55^ and citations therein). We can speculate that *SQLE* and *RAD21* concomitant overexpression would enable a cancer cell to proliferate more effectively through a more proficient DNA damage repair system while dividing (through RAD21), and at the same time to speed up the process by more efficient membrane synthesis (courtesy of SQLE). However, the key point of our research is another: even if other genes found in the same physical region of *SQLE* have their own roles in determining an aggressive cancer phenotype, we have also showed experimentally that SQLE is an attractive *bona fide* target for treatment, while other co-located proteins (such as RAD21) are not readily druggable, and hence less clinically relevant to the purpose of finding novel therapeutic targets in breast cancer and other tumor types.

We have to acknowledge that our *in vitro* experiments are far from exhaustive: the potential for off-targets in pharmacological SQLE inhibition experiments is still present, although terbinafine blocks the activity of mammalian SQLE, and albeit selective, *SQLE* silencing could result in a cytostatic rather than cytotoxic effect on cell lines. Nonetheless, the independent nature of the two methods we used to block SQLE activity (i.e. using a chemical compound known to target also mammalian SQLE and decreasing *SQLE* transcription by siRNA) corroborates the proof of concept that the detrimental biological effects we observed in breast cancer cell lines are indeed likely to result from the selective inhibition of SQLE activity. Moreover, the *SQLE* copy-dosage correlation with terbinafine activity is *per se* highly suggestive of an on-target biological phenomenon, worth of further investigation. Copy dosage alone does not explain entirely the sensitivity to SQLE inhibition in our cell set: in the case of Hs 578T, terbinafine effect is elevated in spite of a relatively low copy dosage, while SK-BR-3 exhibit lower sensitivity to terbinafine than MCF-7, while having an even higher copy number in the *SQLE* region. However, the first cell line exhibits the highest *SQLE* expression of the six we tested, possibly driven by epigenetic mechanisms worth of further investigation, whereas partial resistance phenomena and other post-translational modifications^7^ still to be tested could account for the discrepancy observed in the second line. Finally, SQLE inhibition may be toxic in *in vitro*, but not *in vivo* experiments, due to the limited supply of cholesterol in the employed media: however, fetal bovine serum provides on average 310 μg/mL of such molecule^56^, thus proving cultivated cancer cell lines with an external source of cholesterol upon *de novo* synthesis inhibition.

We are not the first investigators to point out that *SQLE* may have a biological relevance in breast cancer. Other researchers observed *SQLE* overexpression in an adverse prognosis group of ER + , stage I/II breast cancer cases^33^, described aberrant methylation patterns in the 8q12.1-q24.22 genomic region chromosomal region^35^, or reported that *SQLE* amplification and increased transcription was enriched (together with other genes) in a distinctive cluster of triple negative breast tumors or in specific ethnic populations^25^,^34^. Again, we have to remark that we provided here the first systematic, large-scale assessment of *SQLE* CN amplification and overexpression in breast cancer and other tumor types in humans. Our analyses have allowed us to identify the tendency of aggressive breast tumors, especially of the Her2 positive, higher grade ones, to overexpress *SQLE*, and its independent role in determining an unfavorable outcome in breast neoplasms. Finally, to the best of our knowledge no one had so far explored the potential anti-cancer properties of SQLE inhibition in *SQLE*-amplified breast cancer models.

To conclude, we believe our present research has shed light on a neglected metabolic cancer gene by amplification in breast cancer, and possibly in other tumor types. Our findings may thus pave the way to additional studies in the clinical setting, to assess the relevance of SQLE inhibition as a novel cancer treatment option.

## Methods

### Datasets

GISTIC aberration calls, *log2* ratio CN intensities, and RNA-sequencing pre-processed GE intensities generated by the TCGA were downloaded from the Broad Institute TCGA GDAC repository (version 2015/02/04). Only datasets with more than 100 collected cases were considered in our analyses, for a total of 8,783 cases and 22 cancer types. The METABRIC dataset was downloaded from the Sage Bionetworks Synapse repository (last accessed, March 3^rd^, 2015). In our analyses, we only considered patients from the METABRIC dataset with complete information for age, tumor size, NPLN (categorized as 0, 1–3, 4 or more), histological grade, ER and Her2 status by bimodal gene expression assessment, as well as follow-up status. We also excluded cases with no intrinsic subtype classification, as well as those classified as “normal-like”, in light of the controversies that this class of breast cancers may be reflective of too low a cellularity to obtain meaningful CN and GE data^57^. We were therefore left with 1,633 patients to conduct our downstream tests. For aberrations in the chromosome 8q24.13-q24.21 region (whose *log2* ratios were calculated as the means of *SQLE* and *MYC* loci values), we categorized intensities as “gains” if the *log2* ratio for a given sample was > 0.32 and ≤ 0.81, and as “amplifications” if > 0.81. Those empirical thresholds would correspond to three and five gene copies respectively, in a fully clonal region of a near-diploid cancer genome with ~ 50% tumor cell fraction (see formula (1) reported below).

### Cell Lines and Reagents

Cell lines were purchased from ATCC (LGC Standards S.r.l., Milan, Italy), except T-47D and MDA-MB-468 that were obtained from the Developmental Therapeutics Program of the National Cancer Institute (Frederick, MD). MCF-7, T-47D, MDA-MB-468, and MDA-MB-231 cell lines were maintained in RPMI 1640 medium supplemented with 10% FBS, penicillin (50 units/ml), and streptomycin (50 μg/ml) (Life Technologies, Italy). Hs 578T cell line was maintained in DMEM medium supplemented with 10% FBS, insulin (0.01 mg/ml), penicillin (50 units/ml), and streptomycin (50 μg/ml) (Life Technologies, Italy). SK-BR-3 cell line was maintained in McCoy’s 5A medium supplemented with 10% FBS, penicillin (50 units/ml), and streptomycin (50 μg/ml) (Life Technologies, Italy). Terbinafine Hydrochloride CSR was purchased from Sigma-Aldrich, Italy. ON-TARGETplus siRNA kits were purchased from Dharmacon, Thermo Fisher Scientific Inc., Italy. Taqman® Gene Expression Assays and TaqMan® Universal PCR Master Mix were purchased from Life Technologies, Italy.

### Array-CGH experiments

DNA from cell lines was extracted using DNeasy Blood & Tissue Kit (Qiagen GmbH, Hilden, Germany). DNA samples were diluted in 200 μL Nuclease-Free water according to the Manufacturer’s instructions. DNA was quantified with a NanoDrop® ND-1000 spectrophotometer (Thermo Scientific, Inc.). DNA copy number aberrations were determined using high-resolution arrays (SurePrint G3 Human CGH Microarrays, 4 × 180K) (Agilent Technologies, Palo Alto, CA, USA). For DNA labeling and assessment of DNA labeling efficiency, 0.8 μg of amplified test and reference DNA (female normal genomic DNA from Promega, Madison,WI) were labeled using Sure Tag DNA labeling kit (Agilent Technologies, Palo Alto, CA, USA) with Cy5-dUTP and Cy3-dUTP respectively, according to the CGH Enzymatic Labeling Kit Protocol v.7.3 (Agilent Technologies, Palo Alto, CA, USA). Unincorporated nucleotides were then removed using centrifugal filters (Amicon Ultra 0.5ml, Merck Millipore, Merck KGaA, Darmstadt, Germany) according to the Manufacturer’s protocol. Quality analysis and quantification of labeled DNA were performed by NanoDrop® ND-1000 (Thermo Scientific, Inc.) spectrophotometer, measuring A260 (for DNA), A550 (for Cy5) and A649 (for Cy3) to evaluate yield, degree of labeling, and specific activity. To perform array hybridization and scanning, Cy5-labeled DNA from cell lines was mixed with an equivalent amount of Cy3-labeled reference DNA. Repetitive sequences were blocked with human Cot-1 DNA (Invitrogen^TM^, Thermo Scientific, Inc.) and samples were hybridized with Oligo aCGH/ChIP-on-chip Hybridization Kit onto the microarray slides, according to the Manufacturer’s specifications. Following hybridization at 65 °C for 24 h in a rotating oven (Agilent Technologies, Palo Alto, CA, USA) at 20 rpm, slides were washed and scanned using a Agilent Microarray Scanner (G2505C). Resulting images were then elaborated and quality-checked using the Feature Extraction software v11.01.1 (Agilent Technologies, Palo Alto, CA, USA), and exported into .txt files for further analyses.

### Cell viability assays

Five thousand MCF-7, 3.5 × 10^3^ MDA-MB-231, 3.8 × 10^3^ MDA-MB-468, 5 × 10^3^ T-47D, 4.5 × 10^3^ SK-BR-3 or 3.8 × 10^3 ^ Hs 578T cells were plated, in quintuplicate, in 96-well plates, and let to adhere overnight. Cells were then treated with terbinafine in the range 250 μM-250 nM. 72 h later, viability was determined by a colorimetric assay using CellTiter 96® Aqueous.

### Sulforhodamine B assays

Forty-five thousand MCF-7, 2.5 × 10^4 ^MDA-MB-231, 3.5 × 10^4^ MDA-MB-468, 4.5 × 10^4 ^ T-47D, 4.5 × 10^4^ SK-BR-3, or 4.5 × 10^4^ Hs 578T cells were plated in 6-well plates and let to adhere overnight. Cells were treated with 62.5 μM terbinafine for 72 h, then plates were fixed with cold 50% trichloroacetic acid at 4 °C for 30 minutes, washed with cold water and dried overnight. Plates were stained with 0.4% sulforhodamine B (SRB) in 1% acetic acid, gently shaken for 10 minutes, washed with 1% acetic acid to remove unbound stain, dried overnight and photographed. SRB was then dissolved in 10 mM Tris, and optical density was measured at 515 nm on a Tecan Infinite F200 Pro^TM^ plate reader.

### siRNA transfections

Transient transfection of cells was performed using ON-TARGET-Plus Smart Pool SQLE siRNAs, non-targeting control siRNAs and GAPDH positive control siRNAs. Twenty thousand MCF-7 and T-47D, 1.2 × 10^5^ MDA-MB-231 and Hs 578T cells/well were plated in 6-well plates, allowed to adhere for 24 h, and then transfected with a final concentration of 100 nM siRNA/well using Dharmafect® according to the Manufacturer’s instructions. 24 h after transfection, cells were plated for SRB based doubling time measurement and RNA isolation. SQLE silencing was verified by qPCR.

### Doubling time measurements

After siRNA transfection, MCF-7, T-47D, MDA-MB-231, and Hs 578T cells were plated in 24-well plates and allowed to adhere for 24 h. At different time points (0, 15, 25 and 43 h) cells were fixed and analyzed with the SRB method (see above) and optical density was measured at 515 nm on a Tecan Infinite F200 Pro^TM^ plate reader. Doubling time was finally calculated from the signal corresponding to the different time points using the online tool Roth V. 2006 (http://www.doubling-time.com/compute.php).

### qPCR

Total RNA was extracted from cells using the RNeasy minikit (Qiagen S.r.l., Milan, Italy) according to the Manufacturer’s specifications. Concentration and integrity were checked using an Agilent 2100 Bioanalyzer system (Agilent Technologies, Palo Alto, CA, USA). One μg of RNA was reverse-transcribed in a final volume of 50 μl using the High Capacity cDNA Reverse Transcription kit (Invitrogen). 5 μl of the resulting cDNA was used for qPCR, performed in triplicates using a 7900 HT Fast real-time PCR system (Applied Biosystems by Invitrogen) with TaqMan® Gene Expression Assays for human *RPLP0* (Hs99999902_m1), *GAPDH* (Hs99999905_m1), *SQLE* (Hs01123768_m1), and TaqMan® Universal PCR Master Mix. *SQLE* GE was normalized to housekeeping GE (geometric mean of *GAPDH* and *RPLP0*). Comparisons in GE were calculated using the 2^−ΔΔCt^ method.

### Array-CGH data analyses

*Log10* ratios from Agilent feature extraction .txt files were imported in R (http://www.R-project.org/) using the *data.table* package, averaged over probe replicates using the *limma* BioConductor package^58^, back-transformed into linear scale before converting into *log2* ratio data space. After mapping probe location to the NCBI37/hg19 build of the human genome using the UCSC liftOver utility (https://genome.ucsc.edu/cgi-bin/hgLiftOver), data were preprocessed by outlier winsorization with the *copynumber* package^59^ using default options, and segmented by penalized least square regression using a heuristically chosen value of γ = 100, which optimized the number of segments per sample, while not leading to excessive information loss. Segmented data were then used to compute cancer cell line mean ploidy and absolute copy number values for SQLE using ABSOLUTE^60^ with the *copy_num_type* argument set to “*total*”, and following the recommendations from the companion website (http://www.broadinstitute.org/cancer/cga/ABSOLUTE).

Once the most likely ploidy and predominant clone cell fraction for a given cell line was selected, the absolute copy number of *SQLE* locus could be calculated solving the following equation for 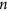:


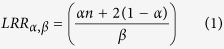


where 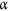 is the predominant clone fraction in a cell mixture, 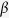 is the mean ploidy returned by ABSOLUTE, 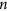 is the integer copy number of a segment, and 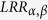 is the measured *log2* ratio for that segment, given α and β. Raw and *log2* normalized data are available in GEO (www.ncbi.nih.gov/projects/geo) under the accession number GSE71395.

### Statistics

Continuous-association tests were carried out using the Spearman’s correlation coefficient, which does not hold *a priori* assumptions over the distribution of the data (normality being systematically violated by *log2* ratio CN measures). FDRs were calculated with the Benjamini-Hochberg multiple testing correction. MCA was performed using the package *FactoMineR* (http://factominer.free.fr/contact/index.html), after categorizing *SQLE* expression intensities in tertiles (as for *MYC*) in the METABRIC dataset, and results were represented with *ggplot2*^61^. Linear regression was employed to analyze the associations of multiple variables with SQLE expression, whereas t tests or one-way ANOVA tests were used for comparison of SQLE expression between categorical variables with two or more levels respectively, using the Tukey honest significant difference method to compare levels in the latter case. Survival curves were plotted using the Kaplan-Meier estimators, generated with the package *survcomp*^62^, and P values were calculated with the log-rank test. For survival analyses including more than one variable, a stepwise backward-forward Cox proportional hazards regression model was employed, starting from all clinical and pathological variables described above, as well as *MYC* and *SQLE* expression levels categorized in tertiles, until minimization of the Akaike Information Criterion was achieved (package MASS^63^). For Cox regression, the P value was calculated using the Wald test. The forest plot in Fig. 3D was generated using the package *rms*. All the aforementioned analyses were conducted in R, as were the related figures. For terbinafine experiments, Hill slope curves, IC_50_ concentrations, and 95% CIs were calculated by fitting a nonlinear function to the quintuplicate data points, whereas silencing efficiency on cell lines was tested using one-way ANOVA with contrasts, followed by the Bonferroni correction for multiple testing. These tests and the corresponding plots were generated using GraphPad PRISM 6. All statistical tests were two-tailed, and null-hypotheses were rejected with P values <0.05. Adobe Illustrator CS6 was used to finalize the illustrations. No data was altered for graphical representation.

## Additional Information

**Accession codes:** Raw and *log2* normalized data for array-CGH experiments are available in GEO (www.ncbi.nih.gov/projects/geo) under the accession number GSE71395.

**How to cite this article**: Brown, D. N. *et al.* Squalene epoxidase is a *bona fide* oncogene by amplification with clinical relevance in breast cancer. *Sci. Rep.* **6**, 19435; doi: 10.1038/srep19435 (2016).

## Supplementary Material

Supplementary Information

## Figures and Tables

**Table 1:** TCGA cancer sets and *SQLE* CN/GE.

Set	N	Loss^A^	Gain^A^	ρ^B^	P	FDR
BRCA	1178	0.03	0.62	0.71	1.71E-181	<10E-6
OV	258	0.05	0.76	0.71	3.43E-41	<10E-6
COAD	323	0.02	0.57	0.61	9.94E-34	<10E-6
KIRP	319	0.03	0.09	0.54	1.29E-25	<10E-6
BLCA	419	0.05	0.62	0.52	7.35E-31	<10E-6
TGCT	150	0.05	0.71	0.52	0.00E + 00	<10E-6
LIHC	411	0.04	0.63	0.49	1.64E-26	<10E-6
LUSC	548	0.04	0.68	0.47	6.91E-31	<10E-6
PAAD	181	0.03	0.37	0.47	2.17E-11	<10E-6
UCEC	196	0.02	0.36	0.46	1.98E-11	<10E-6
CESC	295	0.06	0.4	0.43	2.22E-14	<10E-6
LUAD	560	0.05	0.61	0.41	8.53E-24	<10E-6
SARC	260	0.16	0.31	0.4	2.41E-11	<10E-6
SKCM	470	0.06	0.51	0.39	8.02E-19	<10E-6
HNSC	554	0.01	0.73	0.35	1.13E-17	<10E-6
GBM	158	0.08	0.12	0.3	1.21E-04	<0.001
LAML	157	0	0.13	0.3	1.04E-04	<0.001
PRAD	540	0.01	0.31	0.26	4.72E-10	<10E-6
LGG	526	0.02	0.2	0.22	1.94E-07	<10E-5
KIRC	595	0.09	0.15	0.19	3.19E-06	<10E-5
THCA	564	0.01	0.01	−0.03	5.50E-01	0.5495
THYM	121	0.02	0.09	−0.08	4.05E-01	0.4244

^A^*SQLE* fraction of losses and gains defined by GISTIC 2.0. ^B^Spearman’s correlation coefficient. Full definitions of the TCGA acronyms can be found at https://tcga-data.nci.nih.gov/tcga.

**Table 2:** Multiple linear regression of clinical and pathological variables associated with *SQLE* expression in breast cancer.

Variable		OddsRatio	95% CI^A^	P
Age	<50 vs. 50–64 years old	1.01	0.91–1.13	0.8571
	65 or > vs. 50–64 years old	0.99	0.90–1.08	0.7183
T size	T2 or > vs. T1	1.07	0.99–1.17	0.0889 °
NPLN	1–3 vs. 0	1.08	0.99–1.17	0.0895 °
	4 or > vs. 0	1.03	0.92–1.15	0.5810
Grade	High vs. Low/Intermediate	1.54	1.42–1.67	<2 × 10^−16^***
ER status	Positive vs. Negative	0.92	0.83–1.02	0.1055
Her2 status	Positive vs. Negative	1.29	1.15–1.45	1.9 × 10^−5^ ***

^A^Confidence interval. NPLN: Number of Positive Lymph Nodes. Significance codes: 0 ‘***’ 0.001 ‘**’ 0.01 ‘*’ 0.05 ‘°’ 0.1.

**Table 3:** Cox proportional hazards multiple regression, with overall survival as outcome variable.

Variable		Hazard Ratio	95% CI	P
*SQLE GE*	High vs. Low	1.32	1.03–1.68	0.0267 *
	Intermediate vs. Low	1.10	0.86–1.42	0.4527
Age	<50 vs. 50–64 years–old	1.17	0.91–1.51	0.2402
	65 or > vs. 50–64 years–old	1.31	1.04–1.64	0.0199 *
T size	T2 or > vs. T1	1.45	1.13–1.84	0.0028 **
NPLN	1–3 vs. 0	1.66	1.32–2.09	1.43 × 10^−5^ ***
	4 or > vs. 0	3.47	2.71–4.43	<2 × 10^−16^ ***
Grade	High vs. Low/Intermediate	1.19	0.96–1.48	0.1155
ER status	Positive vs. Negative	0.71	0.56–0.91	0.0064 **
Her2 status	Positive vs. Negative	1.58	1.23–2.02	0.0003 ***

Significance codes: 0 ‘***’ 0.001 ‘**’ 0.01 ‘*’ 0.05.

**Table 4:** Breast cancer cell line *SQLE* characteristics and experimental results.

Cell line	Mean ploidy	CN^A^ / focal^B^	GE^C^ ± 95% CI	IC_50_[μM]^D^ ± 95% CI	DT^E^-hours (scrambled^F^) ± 95% CI	DT - hours (siSQLE^G^)
MCF-7	3.57	9/yes	0.94 ± 0.03	32.1 ± 3.1	61.7 ± 3.7	90.4 ± 5.6
Hs 578T	2.45	5/no	2.02 ± 0.14	52.2 ± 4.0	146.8 ± 5.8	170.3 ± 5.5
SK-BR-3	3.85	13/yes	0.92 ± 0.21	84.9 ± 6.8	n.p.^H^	n.p.
MDA-MB-468	2.79	4/no	1.28 ± 0.02	115.4 ± 10.3	n.p.	n.p.
T-47D	2.62	4/no	0.47 ± 0.02	140.8 ± 18.4	55.3 ± 1.3	73.5 ± 1.2
MDA-MB-231	2.64	1/yes	0.05 ± 0.01	220.9 ± 8.8	48.3 ± 4.2	48.7 ± 2.0

^A^Copy number. ^B^Defined thus when aberration encompasses less than 20% of chromosomal arm. ^C^Gene expression. ^D^Terbinafine micromolar concentration inhibiting cellular growth by 50%. ^E^Doubling time. ^F^Negative siRNA control. ^G^*SQLE*-targeting siRNA. ^H^Not performed.
