# Supplementary material for: Squalene epoxidase is a bona fide oncogene by amplification with clinical relevance in breast cancer
Source: Sci Rep. 2016 Jan 18;6:19435. doi: 10.1038/srep19435 (PMC4726025; doi:10.1038/srep19435)
Supplement: Supplementary Information [file srep19435-s1.pdf]

Title:

Squalene epoxidase is a *bona fide* oncogene by amplification with clinical relevance in breast cancer.

Authors:

David N Brown, Irene Caffa, Gabriella Cirmena, Daniela Piras, Anna Garuti, Maurizio Gallo, Saverio Alberti, Alessio Nencioni, Alberto Ballestrero, Gabriele Zoppoli.

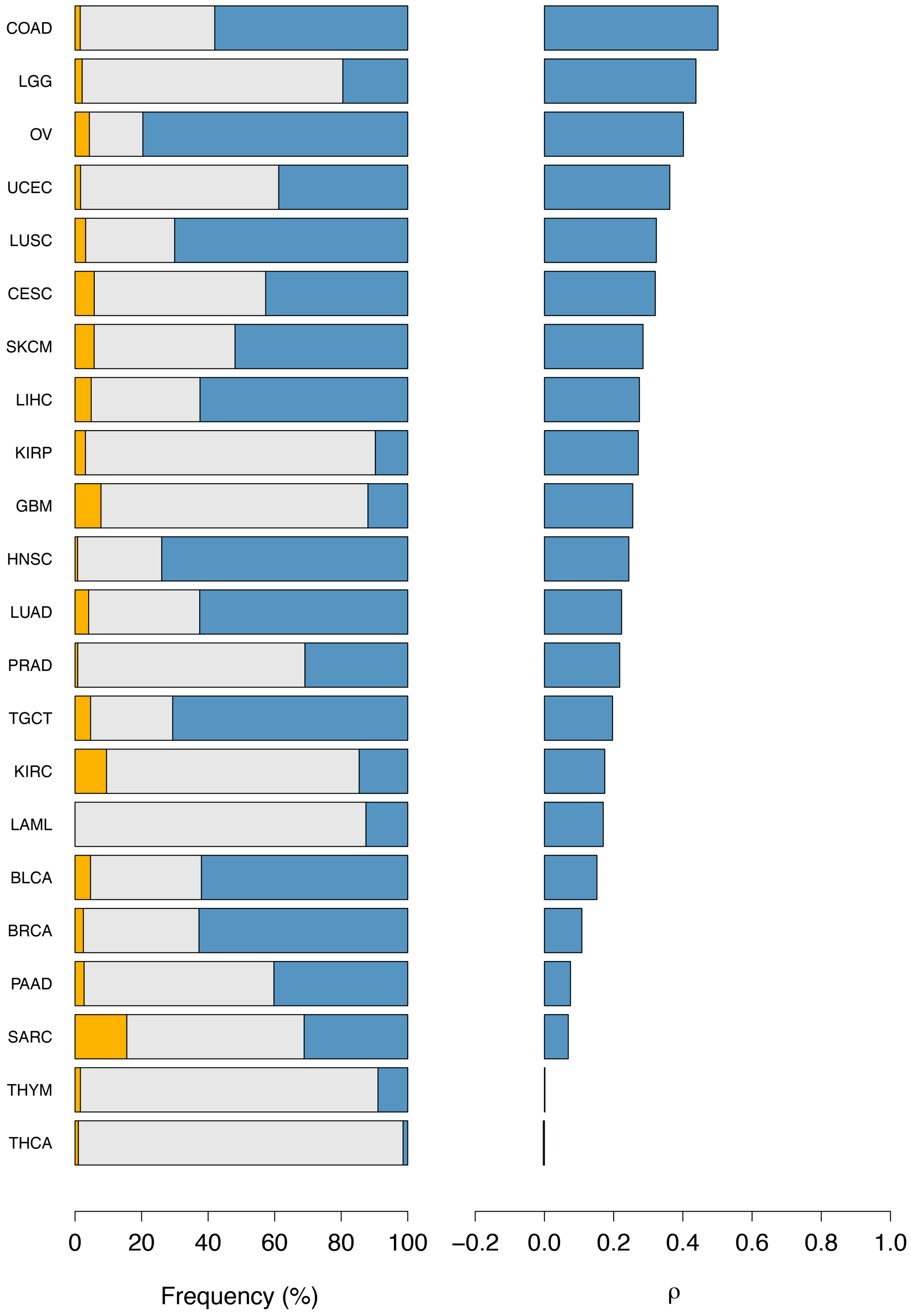
COAD
LGG
OV
UCEC
LUSC
CESC
SKCM
LIHC
KIRP
GBM
HNSC
LUAD
PRAD
TGCT
KIRC
LAML
BLCA
BRCA
PAAD
SARC
THYM
THCA
0
20
40
60
80
100
Frequency (%)
−0.2
0.0
0.2
0.4
0.6
0.8
1.0
ρ

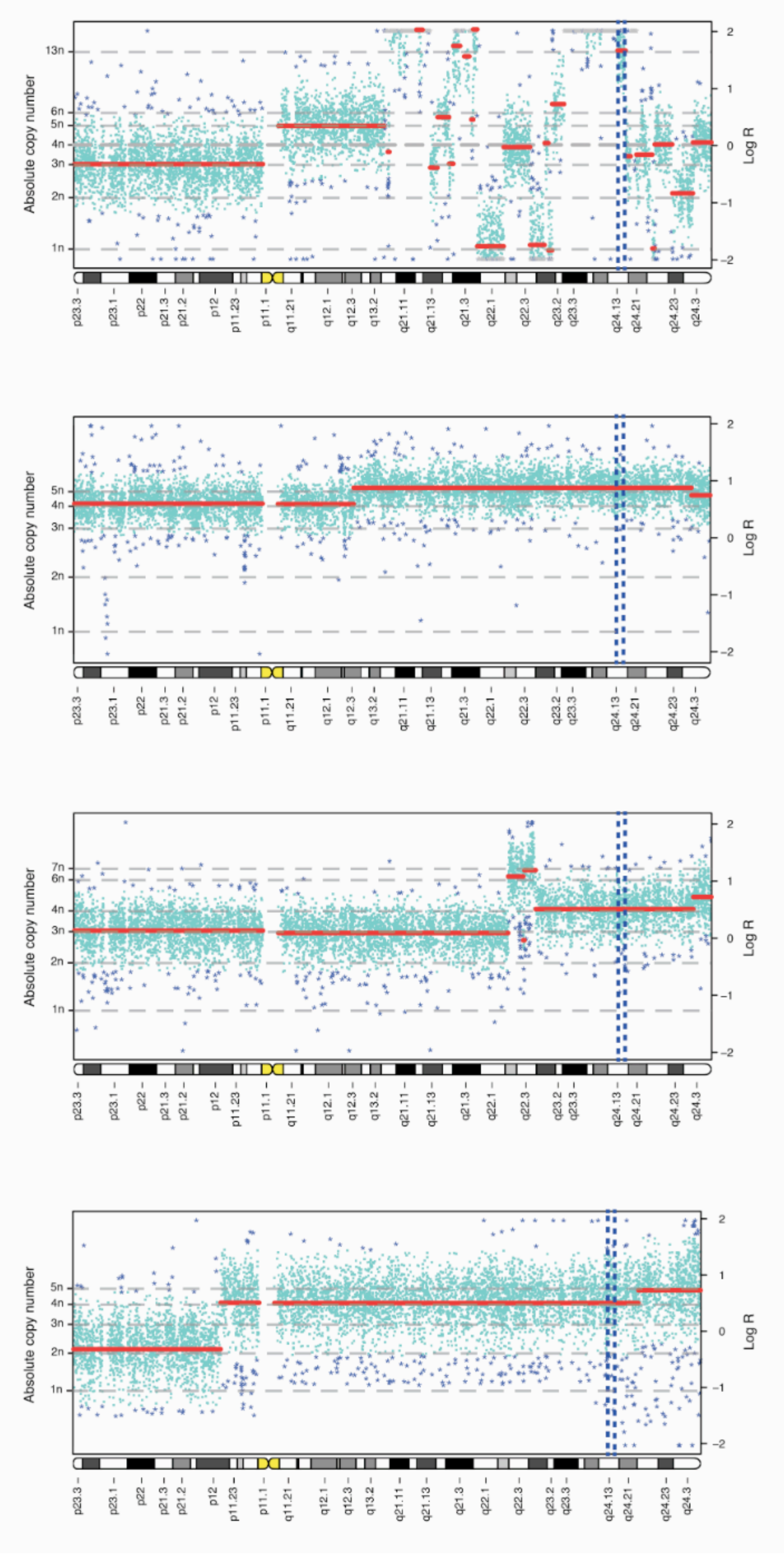

Absolute copy number
Log R
13n
6n
5n
4n
3n
2n
1n
7n
p23.3
p23.1
p22
p21.3
p21.2
p12
p11.23
p11.1
q11.21
q12.1
q12.3
q13.2
q21.11
q21.13
q21.3
q22.1
q22.3
q23.2
q23.3
q24.13
q24.21
q24.23
q24.3

Set	N	Loss[A]	Gain[A]	ρ^B	P	FDR
COAD	323	0.02	0.58	0.5	5.50E-22	< 10E-6
LGG	526	0.02	0.19	0.44	4.80E-26	< 10E-6
OV	258	0.04	0.8	0.4	2.03E-11	< 10E-6
UCEC	196	0.02	0.39	0.36	1.81E-07	< 10E-5
LUSC	548	0.03	0.7	0.32	8.10E-15	< 10E-6
CESC	295	0.06	0.43	0.32	1.82E-08	< 10E-6
SKCM	470	0.06	0.52	0.28	3.14E-10	< 10E-6
LIHC	411	0.05	0.62	0.27	1.54E-08	< 10E-6
KIRP	319	0.03	0.1	0.27	8.60E-07	<10E-4
GBM	158	0.08	0.12	0.26	1.21E-03	< 0.001
HNSC	554	0.01	0.74	0.24	5.98E-09	< 10E-6
LUAD	560	0.04	0.63	0.22	9.63E-08	< 10E-5
PRAD	540	0.01	0.31	0.22	3.28E-07	< 10E-5
TGCT	150	0.05	0.71	0.2	1.57E-02	<0.05
KIRC	595	0.09	0.15	0.17	1.89E-05	< 0.0001
LAML	157	0	0.13	0.17	3.33E-02	<0.05
BLCA	419	0.05	0.62	0.15	1.84E-03	<0.01
BRCA	1178	0.03	0.63	0.11	1.95E-04	< 0.001
PAAD	181	0.03	0.4	0.08	3.14E-01	0.3452
SARC	260	0.16	0.31	0.07	2.68E-01	0.3106
THCA	564	0.01	0.01	0	9.37E-01	0.9817
THYM	121	0.02	0.09	0	9.88E-01	0.9876

Doubling time measurements (h)		
	**siRNA Scramble**	**siRNA SQLE**
MCF-7	56,5+/-2,6	98,5+/-3,4
Hs578T	134,6+/-2,7	178,2+/-2,6
T47D	55,5+/-3,1	115,9+/-4,2
MDA-MB231	42,2+/-3,2	42,9+/-4,1

Terbinafine titration					
	**IC50**				**IC50**
MCF-7	46,5uM			MDA-MB 468	105uM
Hs578T	64,05uM			T47D	123,6uM
SKBR-3	91,4uM			MDA-MB 231	211,9uM

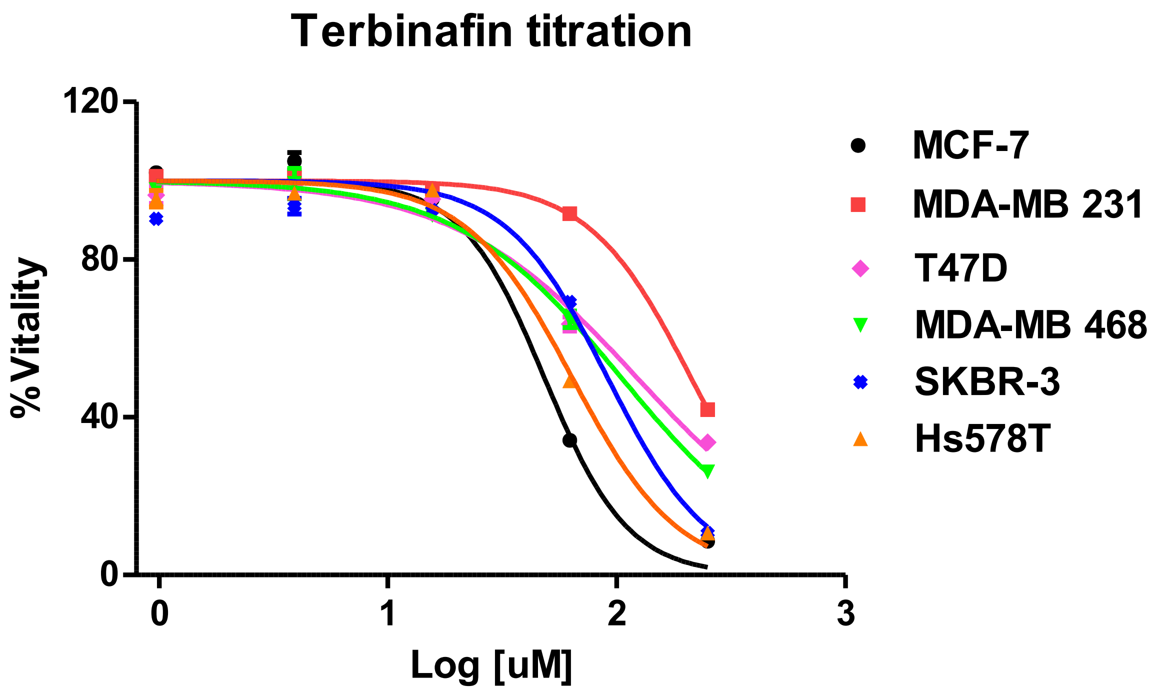

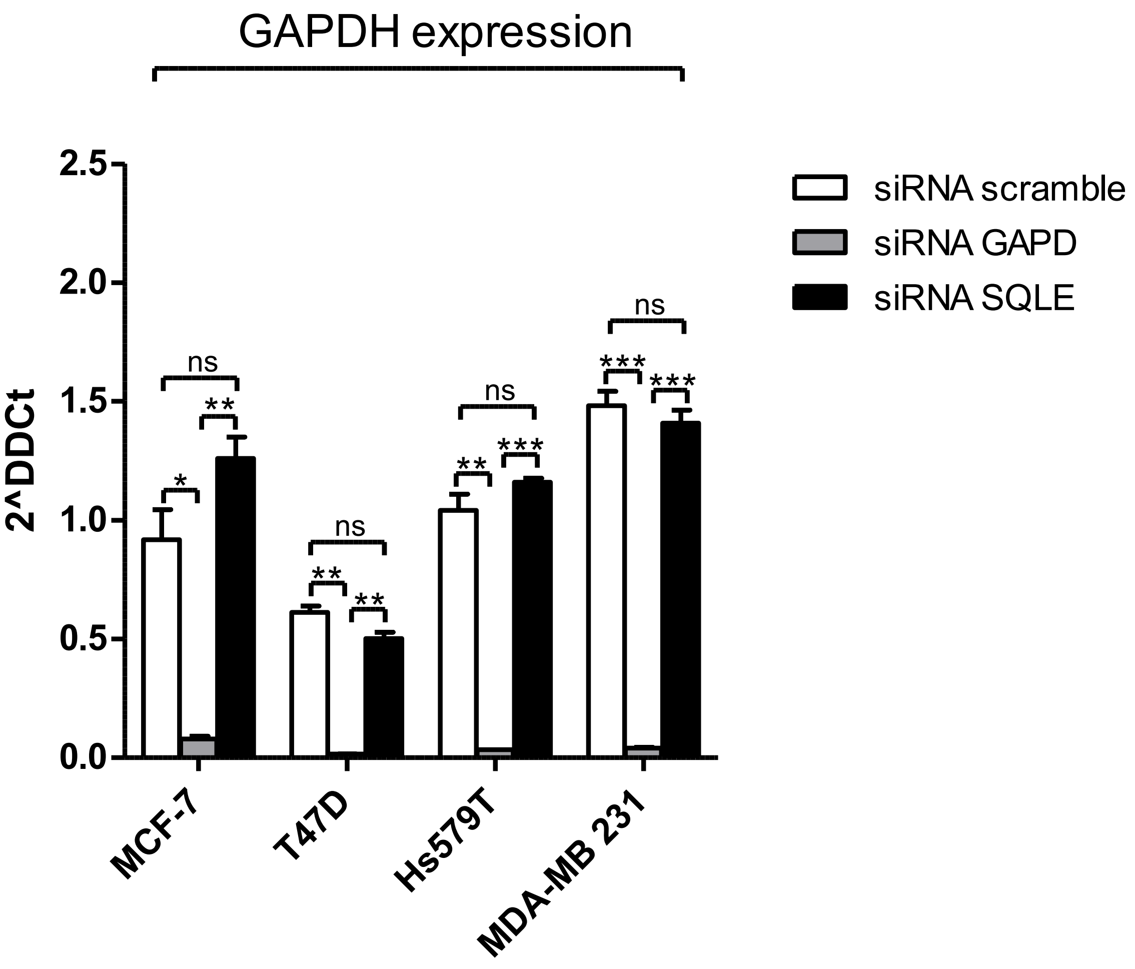
GAPDH expression
2^DDCt
2.5
2.0
1.5
1.0
0.5
0.0
ns
*
**
***
MCF-7
T47D
Hs579T
MDA-MB 231
siRNA scramble
siRNA GAPD
siRNA SQLE

Supplemental figure 1 (page 2): **Correlation of MYC CN and GE in TCGA.** All cancer histotypes with at least 100 cases collected by the TCGA were assessed for the presence of MYC copy number gains and losses (blue and orange respectively, left bar chart), and *log2* ratios were correlated with normalized MYC gene expression using the Spearman's correlation coefficient (ρ value on the *x*-axis, decreasing from highest to lowest, right bar chart). Full definitions of the TCGA acronyms can be found at https://tcga-data.nci.nih.gov/tcga.

Supplemental figure 2 (page 3): **Chromosome 8 karyogram of (top to bottom) SK-BR-3, Hs 578T, MDA-MB-468, and T-47D.** Left *y*-axis: absolute copy numbers. Right *y*-axis: *log2* ratio. *x*-axis: chromosome 8 cytobands. Thick horizontal red lines represent segmented regions, turquoise dots are individual probes, whereas blue asterisks are outlier probes excluded from segmentation analysis. Dashed blue boxes enclose the SQLE locus.

Supplementary Table S1 (page 4): **TCGA cancer sets and MYC CN/GE.** [A]SQLE fraction of losses and gains defined by GISTIC 2.0. [B]Spearman's correlation coefficient. Full definitions of the TCGA acronyms can be found at https://tcga-data.nci.nih.gov/tcga.

Supplementary Table S2 (page 5): **Second biological replicate of siRNA doubling time measurements in hours.** Time is expressed in hours (h) for doubling time of cell lines treated with scramble or targeting siRNA.

Supplementary figure 3 (page 6): **Second biological replicate of terbinafine titration in the six-cell line set.** IC50 = terbinafine concentration inhibiting 50% of cell growth. uM = micromolar concentration. *y*-axis: Percent of cells viable by MTT assay, *x*-axis: micromolar concentration of terbinafine. Whiskers represent standard error of the means (S.E.M.).

Supplementary figure 4 (page 7): **GAPDH expression in scramble, GAPDH, and SQLE siRNA treated cells.** **= $P < 0.01$, *** = $P < 0.001$, NS = non significant. Whiskers represent S.E.M. P values are calculated by ANOVA with contrasts with the Bonferroni correction for multiple testing. Data are normalized using the median of scramble siRNA.
